# Adopting Best Practices from Team Science in a Healthcare Improvement Research Network: The Impact on Dissemination and Implementation

**DOI:** 10.1155/2013/814360

**Published:** 2013-03-17

**Authors:** Frank Puga, Kathleen R. Stevens, Darpan I. Patel

**Affiliations:** Academic Center for Evidence-Based Practice, School of Nursing, University of Texas Health Science Center San Antonio, MSC 7949, 7703 Floyd Curl Drive, San Antonio, TX 78229, USA

## Abstract

Healthcare is a complex adaptive system, and efforts to improve through the implementation of best practice are well served by various interacting disciplines within the system. As a transdisciplinary model is new to clinicians, an infrastructure that creates academic-practice partnerships and builds capacity for scientific collaboration is necessary to test, spread, and implement improvement strategies. This paper describes the adoption of best practices from the science of team science in a healthcare improvement research network and the impact on conducting a large-scale network study. Key components of the research network infrastructure were mapped to a team science framework and evaluated in terms of their effectiveness and impact on a national study of nursing operations. Results from this study revealed an effective integration of the team science principles which facilitated the rapid collection of a large dataset. Implications of this study support a collaborative model for improvement research and stress a need for future research and funding to further evaluate the impact on dissemination and implementation.

## 1. Introduction

Healthcare has been described as a complex adaptive system (CAS) that involves multiple, interdependent entities and organizational levels [1–3]. Such complexity poses a challenge for transformative change as relationships within a CAS are nonlinear and unpredictable [4, 5]. This challenge is echoed by leaders in the field of quality improvement who have identified shortcomings, such as a lack of rigorous research methods, a failure to study contextual variables, and weak evaluation designs [6–8]. These shortcomings are an indication that the interwoven processes of healthcare delivery are difficult for a single investigator to tease apart. Thus, a collaborative model that integrates multiple perspectives from several disciplines may help advance the field of improvement science and facilitate dissemination and implementation strategies. 

Transdisciplinary collaboration is a potentially effective model as it brings together a diverse group of individuals who fully integrate theories, methodologies, and frameworks from their respective fields to work as a cohesive unit on complex issues [9]. This differs from multidisciplinary and interdisciplinary collaboration where multiple individuals work together but remain grounded in their respective ideologies [9–11]. Recently, a few studies have addressed the potential of transdisciplinary collaboration to develop effective interventions in healthcare [12–14]. Despite these efforts, research in healthcare improvement has typically involved a single researcher studying an intervention at a single hospital or clinical unit. This approach is ineffective as the complexity of organizational change and contextual influence can impact the implementation and effectiveness of an intervention [7, 15]. 

The current consensus in the field of improvement science is that new methodologies are needed to address complex issues associated with healthcare delivery [6, 16]. Specifically, researchers and clinicians need to adopt strategies that redefine relationships and establish new ways of communicating [6, 16–18]. A transdisciplinary model may be the solution. In fact, implementation science frameworks suggest that transdisciplinary interaction promotes effective and sustainable intervention programs [19]. 

The shift from a single investigator/single site model to a transdisciplinary investigative team/multisite model calls for unique interactions between clinicians and researchers. Currently, there are few competencies and models for team performance; especially in the context of building transdisciplinary teams for improvement [20]. This paper describes the adoption of a transdisciplinary model in a healthcare improvement research network and the impact of collaboration on the conduct of a national improvement study. The results from this study show promise for enhancing research on improvement and uptake of best practices using transdisciplinary collaboration. 

## 2. Theoretical Framework

The guiding framework to implement and evaluate a transdisciplinary collaborative model in this project was grounded in principles from the science of team science (SciTS). SciTS is a hybrid scientific field that incorporates factors that facilitate and hinder scientific collaboration [21]. This field has generated the evidence to support the claim that transdisciplinary collaboration spurs innovation and accelerates scientific discovery [22–24]. Additionally, SciTS is founded on evidence from human factors engineering and offers guidance on how investigative teams can be effective in studying healthcare improvement. 

The SciTS framework for this study was adapted from a previous review of factors contributing to collaborative success [25]. Concepts were adopted by a healthcare research network based on the number of times they were referenced, how often they were used for an evaluation of collaborative work, and their relevance to long-distance collaboration. The four concepts in the framework are as follows: (1) readiness for collaboration, (2) creating a shared mental model, (3) management and planning, and (4) virtual readiness. These concepts are briefly described below. 

### 2.1. Readiness for Collaboration

In order for a collaboration to be successful, scientific teams need to be ready to collaborate on an individual, group, and organizational level [26]. This readiness comes in the form of one's adaptability and flexibility, openness to diverse perspectives, communication skills, conflict resolution, respect for others, institutional support, and availability of reliable technology [27, 28]. Each of these qualities is essential for collaborative processes and group productivity [29].

### 2.2. Shared Mental Model

A shared mental model is the organized knowledge that people use to interpret, explain, analyze, and predict what is happening around them [30]. A shared mental model helps collaborating researchers to coordinate with teammates, form accurate expectations about tasks, and understand and anticipate each other's actions and needs [31]. Teams make fewer mistakes than individuals, especially when each team member knows his or her responsibilities, as well as the responsibilities of other team members [32]. Organizational researchers have observed that when team members have a shared mental model, it increases the overall team engagement and performance [30]. 

### 2.3. Management and Planning

The success of a project is dependent on the way the work is organized and carried out in a scientific team [22]. Structuring and monitoring are necessary to maintain scientific rigor and protocol fidelity especially in multisite studies. Without proper management, the project may not meet its objectives, or results generated from a study may not be reliable due to variations in study implementation and data collection.

### 2.4. Virtual Readiness

Virtual collaboration requires a high quality, well-functioning technical infrastructure that is designed to fit the nature of the work [22, 27]. Research suggests that user-centered technology, such as access to email, web space for sharing documents, and a centralized databases, is essential features for long distance collaboration [25, 33]. Without a stable technical infrastructure to support virtual collaboration, readiness to collaborate, shared mental models, and management planning are ineffective in multisite, transdisciplinary teams.

## 3. Methodology

### 3.1. Design

The main research questions for this study were (1) does an SciTS framework facilitate transdisciplinary collaboration in a healthcare improvement research network? and (2) what impact does a transdisciplinary model have on the conduct of a national, multisite improvement study? Mixed methods were used to collect and analyze data. This project was approved by the University of Texas Health Science Center San Antonio (UTHSCSA) Institutional Review Board (IRB).

### 3.2. Sample and Setting

The objectives of this project focused on transdisciplinary collaboration within a multisite study conducted by the Improvement Science Research Network (ISRN). The ISRN is a national hospital-based research network comprised of approximately 200 partners from academic and practice settings across the nation that have an interest in studying quality improvement. Demographic information for the ISRN membership at the time of this study is presented in Table 1. The majority of the membership consisted of nurses working in various fields in acute care settings or at academic institutions. Doctorate prepared members represented a variety of fields including nursing research, health services research, public health, translational science, quality improvement, and implementation science. 

From the ISRN membership, research partners are invited to join Research Collaboratives for ISRN studies. Each network study is supported by a research infrastructure modeled after practice-based research networks (PBRNs) and multisite clinical trials. The research infrastructure supports a Virtual Collaboratory, or center without walls, that allows a team of scientists to work on common problems regardless of location [25, 34]. The cornerstone of this infrastructure is a set of national research priorities that serve as a common rallying point to attract diverse perspectives and integrate paradigms from multiple disciplines in order to study improvement. These priorities were developed through a national stakeholder survey and include transitions of care, high-performing clinical systems and microsystems approaches to improvement, evidence-based quality improvement and best practice, and learning organizations and culture of quality and safety. 

ISRN studies are supported through a cyber infrastructure equipped with appropriate communications technology (virtual meeting platforms and teleconference lines), a dedicated web space for sharing information and a central database for data entry. This technology platform is backed with sufficient bandwidth, electronic networking capabilities, and technical support. Adequate capacity for data security, integrity, privacy, rapid retrieval, and long-term archiving are also integrated into the ISRN infrastructure to support data entry and storage. 

For this study, data were collected from 14 collaborating hospitals. The average hospital size was 425 beds (range: 120–665) with an average daily census of 74.7% (range: 60%–100%). Each site had at least one principal investigator (PI) and one research coordinator. Both PIs and research coordinators had nursing backgrounds with educational experiences that ranged from Bachelor's to Doctorate levels. Although the collaborative team also consisted of two study PIs (a doctorate-prepared nurse and a physician, both with extensive research backgrounds) and two research scientists (both PhD prepared with backgrounds in psychology and physiology), they were not included in the data collection due to their proximity to the evaluation and interpretation of results.

### 3.3. Procedure

SciTS principles were used to develop the key ISRN resources to facilitate the conduct of network studies. These resources included training meetings to build readiness and a shared mental model, a protocol implementation kit to facilitate study management, and a robust technical infrastructure to support long distance collaboration. Sites on the investigative team were required to complete capacity building exercises, including a training session on participating in a Research Collaborative. This 2-hour training session consisted of an overview of the ISRN mission and research priorities, a review of the study objectives, an introduction to the protocol, an explanation of data entry procedures, and a presentation of how to work as a collaborative team. Sites were also asked to review recorded ISRN presentations from experts in the fields of team science and virtual collaboration. Concepts from these presentations were discussed during study meetings. Finally, sites were given a resource guidebook on building successful research collaboration. This guidebook synthesized essential qualities for succeeding in research collaboratives for healthcare improvement [35].

 To facilitate the study implementation and ensure the protocol fidelity, sites were given a protocol implementation kit (PIK). The PIK was designed with two goals in mind: (1) systematic implementation of the study protocol across multiple sites to yield analyzable/reliable data and valid study outcomes; (2) guidance for site PIs to facilitate the conduct of the study. The PIK provided a structured overview of various topics related to the study including: forming the project team, preparing for IRB submission, establishing project timelines, identifying participating staff members, using data collection materials, submitting data to the ISRN, and understanding results from the study. 

 Finally, sites were given access to a variety of technical resources, including a shared web space, access to conference lines, and a centralized database. A SharePoint site was developed to allow for the easy exchange of study documents. GoToMeeting (Citrix Systems, Inc.; Santa Barbara, CA) and a teleconferencing line were used for real-time meetings and presentations. Lastly, a centralized database was created and maintained by the UTHSCSA Department of Epidemiology and Biostatistics using the Informatics Data Exchange and Acquisition System (IDEAS), a robust web-based human studies research informatics data management framework. 

### 3.4. Data Collection and Analysis

Variables that best represented measures of collaboration readiness, study management, and the presence of a strong mental model were selected for analysis. These variables included (1) the number of protocol deviations (study management), (2) meeting attendance percentage (shared mental model), (3) number of study timeline adjustments (readiness), and (4) number of questions received from PIs and coordinators regarding study objectives (study management, shared mental model, and readiness) which were all collected from regulatory documents (e.g., protocol deviation logs), study meeting notes, and email communication. 

Additionally, site PIs (*N* = 16) and research coordinators (*N* = 14) were surveyed on ISRN coordinating center services and resources. The survey was designed in Survey Monkey (SurveyMonkey.com, LLC; Palo Alto, CA), and a link to access the survey was emailed to Site PIs and research coordinators. Respondents were asked to rate the importance and quality of ISRN features and services on 6-point Likert scale. Dillman's best practices for internet surveys were adopted for data collection [36]. Results on key resources and services for a collaborative environment are reported. Frequencies and percentages were used to summarize raw data. 

## 4. Results

### 4.1. Study Document Review

The number of protocol deviations, number of email correspondence, number of timeline adjustments, and meeting attendance percentage is broken down by site in Table 2. On average, collaborating sites reported six protocol deviations. Examples of deviations reported were study instruments turned in after data collection period ended and data collected from unconsented participants. A review of the study notes revealed that a total of ten sites (70%) successfully adhered to study timelines. Study timeline delays were due to late IRB approvals, accreditation visits, or a change in health information technology systems. The average number of email correspondences per site was 55 emails (range: 20–101 emails). The majority of questions from site PIs and coordinators pertained to the conduct of the study, including IRB regulations, participant recruitment, data collection, and data entry. Few questions (<1%) were directed towards the study's goals or the function of the collaboration. Finally, the attendance on conference calls averaged 94% with sites being absent due to scheduling conflicts or unexpected emergencies. 

### 4.2. ISRN Coordinating Center Effectiveness Survey

A survey on the quality and importance of ISRN coordinating center services and resources was sent to a total 30 site PIs and coordinators. A total of 17 responses were received for response rate of 57%. Demographic information from survey participants is presented in Table 3. Each respondent had previous experience participating in studies as a part of a formal investigative team (mean: 6 studies per respondent, Range: 1–20 studies). On average, half of these studies involved professions other than nursing (mean: 3 studies per respondent, range: 0–15 studies). Results from survey items specific to providing a collaborative environment are presented in Table 4. Most services and resources were positively rated in terms of quality, but those related to organizational structure, study objectives, and communication were rated highest by respondents (>90%). 

## 5. Discussion

The present study described the adoption of a transdisciplinary collaborative model in a multisite improvement study. Resources developed using best practices from SciTS facilitated the study's conduct as evident by consistent protocol implementation, active engagement, and focused task completion. The impact on productivity could have implications on identifying effective improvement strategies that lead to rapid uptake and spread. Improvement scientists and clinicians in the field need to engage in systems change to support team science and adopt resources, such as research collaborative guidebooks and training modules on SciTS principles, to build capacity for transdisciplinary collaboration.

In the context of collaboration readiness, individuals who demonstrate high levels of readiness are less likely to make mistakes, communicate effectively, and complete objectives in a timely manner [29]. This level of readiness can create a fertile environment for multiple disciplines to come together, blend knowledge, and create a new intellectual space [9]. Both researchers and clinicians must raise their readiness capacity in order to spur transdisciplinary initiatives in the field. The importance of this concept is further validated by the development of standardized evaluations that directly assess readiness in scientific teams, including the National Cancer Institute's Transdisciplinary Research on Energetics and Cancer (TREC) survey and the Collaboration Success Wizard [29, 37].

Additionally, resources are needed to cultivate shared mental models in the field. Currently resources do exist such as national research priorities in improvement science, frameworks for implementation science, and national methodology conferences [6, 15, 38–41]; yet, more is needed to help build transdisciplinary scientific teams for research. For example, experts have called for the establishment of professional organization for improvement and implementation science [42]. Attention has also been called to taxonomy development for an overarching language in hybrid fields [43]. In the present study, the ISRN research priorities and mission were intended to help create a shared mental model that kept research partners engaged in conducting the study. Without a mental model, the observed level of engagement would have decreased and impacted the quality of study outcomes.

The present study also demonstrated the effectiveness of the ISRN research infrastructure, in particular, the management of a multisite study with standardized approaches. A protocol implementation kit was shown to be an effective tool for ensuring protocol fidelity. The use of such a tool helps meet a need in the field for rigorous research and standardized implementation methods [6, 16, 39]. A failure to ensure consistent implementation and protocol fidelity can decrease the reliability of research findings and thus impact translation into multiple clinical settings. 

The move from research to clinical practice requires scaling-up quality improvement initiatives to large-scale network studies. Doing so may yield more effective improvement and implementation strategies, improve dissemination of knowledge, and ultimately change policy. An example of transdisciplinary collaboration is in the numbers associated with the ISRN study evaluated for this paper. The dataset consisted of 24,014 data points reported by 716 acute care medical-surgical nurses across 14 hospitals. This represented 2,452 day shifts and 1,447 night shifts. A team-based approach enabled this particular study to capture a representative national sample to enhance the quality of research and raise scientific rigor. Such scale up of an improvement study will hopefully affect spread and generalizability.

A weakness of the present study is that team science concepts were measured indirectly. This is an indication of a need for established evaluation tools for transdisciplinary collaboration in improvement and implementation research. In fact, there is a nonhealthcare specific tool that is designed to identify potential barriers to collaboration and provide recommendations for improvement. The Collaboration Success Wizard (CSW) was developed to evaluate virtual collaborations based on 15 years of evidence that has identified factors that predict collaborative success [25]. An external evaluation of the same ISRN study described in the present paper revealed that the project was well positioned for successful collaboration using the CSW [44]. 

The present study was also limited in its ability to measure the impact of transdisciplinary collaboration on dissemination and implementation. This is mostly due to the fact that this type of evaluation requires time. Thus, more research and funding are needed to demonstrate the impact of transdisciplinary research on spread and uptake of evidence-based quality improvement uptake. Given the magnitude of data collected from improvement studies using this approach, it can be inferred that rigors methods and generalizable results may speed uptake and spread; however, data are still needed to justify this claim. 

## 6. Conclusion

This project demonstrated the effectiveness of a transdisciplinary model for academic and clinical scientists that are interested in studying improvement. Indications are that national improvement studies are positively assisted by the guidance gleaned from SciTS. Further research is needed on the causal relationship between team-based research and dissemination and implementation of evidence-based quality improvement. With advances in team research and increased funding opportunities to investigate dissemination, implementation, and improvement strategies in healthcare, the gap between what works and what is actually practiced will narrow to raise the quality of care delivery.

## Figures and Tables

**Table 1:** Demographic breakdown of ISRN membership (total *N* = 194).

Demographic	Category	*N*	% of total membership
	Academic	80	41.2%
Institution	Clinical	78	40.2%
	Not reported	36	18.6%

Membership type	Student	13	6.7%
	Professional	182	93.8%

Education	Bachelor	5	2.6%
	Master	33	17.0%
	Doctorate	86	44.3%
	Not reported	70	36.1%

	Nurse	26	13.4%
	Nurse researcher	22	11.3%
	Physician	3	1.5%
Profession	Educator	24	12.4%
	Faculty	74	38.1%
	Other*	34	17.5%
	Not reported	11	5.7%

*For example, program director, manager, executive, and coordinator.

**Table 2:** Protocol deviations, emails received, and timeline adjustments.

Collaborating sites	Protocol deviations (*N*)	Emails received (*N*)	Timeline adjustment	Meeting attendance (%)
Site 1	2	55	Yes	83.3%
Site 2	NA	104	No	100.0%
Site 3	6	42	No	100.0%
Site 4	NA	75	Yes	100.0%
Site 5	1	39	No	100.0%
Site 6	10	32	Yes	100.0%
Site 7	19	78	No	100.0%
Site 8	2	101	No	100.0%
Site 9	NA	43	Yes	100.0%
Site 10	4	32	No	83.3%
Site 11	NA	81	No	100.0%
Site 12	14	33	No	100.0%
Site 13	0	20	Yes	66.7%
Site 14	15	37	No	83.3%

NA: not available.

**Table 3:** Demographic breakdown of survey respondents (total *N* = 17).

Demographic	Category	*N*	% of respondents
	Bachelor's	1	5.9%
Education	Master's	5	29.4%
	Doctorate	10	58.8%
	Other	1	5.9%

	Frontline	1	5.9%
	Staff development	2	11.8%
Current position	Faculty—academic institution	4	23.5%
	Research scientist— clinically based	8	47.1%
	Administrator	1	5.9%
	Manager	0	0.0%
	Other	1	5.9%

	less than 1 year	0	0.0%
	1 to 5 years	0	0.0%
	6 to 10 years	3	17.6%
Career experience	11 to 15 years	0	0.0%
	16 to 20 years	1	5.9%
	>20 years	13	76.5%
	Not reported	0	0.0%

**Table 4:** Summary on the quality of services offered by the ISRN coordinating center.

ISRN coordinating center services and resources	Quality (% responses)			
	Low	Medium	High	Total
Focused on the improvement science as the network mission.	0	0	100	100
Responded quickly and effectively to the site PIs emails and phone calls.	0	6	94	100
Provided clear description of the structure of ISRN (e.g., network PIs; site PIs; network studies).	0	6	94	100
Established Network structure and processes that supported collaboration.	0	19	81	100
Furnished clear call for letters of intent and application for STAR-2 sites.	0	6	94	100
Supported site PIs and collaborators as full partners in the study.	0	13	88	100
Outlined fair guidelines for collaboration (e.g., publication credits).	0	25	75	100
Provided a useful SharePoint site.	7	13	80	100
Engaged sites in an action plan for continuing in ISRN network studies.	13	20	67	100
